# Effect of Heat-Treated *Lactiplantibacillus plantarum* nF1 on the Immune System Including Natural Killer Cell Activity: A Randomized, Placebo-Controlled, Double-Blind Study

**DOI:** 10.3390/nu16091339

**Published:** 2024-04-29

**Authors:** Geun-Hye Hong, So-Young Lee, In Ah Kim, Jangmi Suk, Chaemin Baeg, Ji Yeon Kim, Sehee Lee, Kyeong Jin Kim, Ki Tae Kim, Min Gee Kim, Kun-Young Park

**Affiliations:** 1 IMMUNOBIOTECH Corp., Seoul 06628, Republic of Korea; ghhong@immunobiotech.co.kr (G.-H.H.); sylee@immunobiotech.co.kr (S.-Y.L.);; 2 Global Medical Research Center, Seoul 03737, Republic of Korea; inah@gmrc.co.kr (I.A.K.); rose@gmrc.co.kr (J.S.); cm3690@gmrc.co.kr (C.B.); 3 Department of Food Science and Biotechnology, Seoul National University of Science and Technology, Seoul 01811, Republic of Korea; jiyeonk@seoultech.ac.kr (J.Y.K.); tpgml3891@seoultech.ac.kr (S.L.); 4 Department of Nano Bio Engineering, Seoul National University of Science and Technology, Seoul 01811, Republic of Korea; jinnykim@seoultech.ac.kr

**Keywords:** immune function, IL-12, NK cell activity, heat-treated *Lactiplantibacillus plantarum* nF1, postbiotics

## Abstract

Heat-treated *Lactiplantibacillus plantarum* nF1 (HT-nF1) increases immune cell activation and the production of various immunomodulators (e.g., interleukin (IL)-12) as well as immunoglobulin (Ig) G, which plays an important role in humoral immunity, and IgA, which activates mucosal immunity. To determine the effect of HT-nF1 intake on improving immune function, a randomized, double-blind, placebo-controlled study was conducted on 100 subjects with normal white blood cell counts. The HT-nF1 group was administered capsules containing 5 × 10^11^ cells of HT-nF1 once a day for 8 weeks. After 8 weeks of HT-nF1 intake, significant changes in IL-12 were observed in the HT-nF1 group (*p* = 0.045). In particular, the change in natural killer (NK) cell activity significantly increased in subjects with low secretory (s) IgA (≤49.61 μg/mL) and low NK activity (E:T = 10:1) (≤3.59%). These results suggest that HT-nF1 has no safety issues and improves the innate immune function by regulating T helper (Th)1-related immune factors. Therefore, we confirmed that HT-nF1 not only has a positive effect on regulating the body’s immunity, but it is also a safe material for the human body, which confirms its potential as a functional health food ingredient.

## 1. Introduction

The immune response is a defense system that maintains homeostasis and plays a very important role in identifying, resisting, and eliminating external pathogens as well as deformed and malignant cells that occur naturally in the body [1]. Natural killer (NK) cells, macrophages, and dendritic cells are involved in this process [2,3]. In particular, NK cells are key factors in innate immunity [4]. NK cells are activated by various cytokines such as interleukin (IL)-2 and IL-12 [5]. They play an important role in increasing the innate immune response to pathogens by secreting interferon (IFN)-γ, as well as activating and maturing monocytes and dendritic cells [6]. In addition, they are known to play an important role during the early stages of the T helper (Th)1 immune response [7]. In particular, IL-12 plays an important role in the immune response by increasing the production of IFN-γ in NK cells and T cells and inducing the proliferation of natural killer cells and the Th1 response [8].

Recently, the importance of health and immunity has been emphasized because of the spread of infectious diseases such as COVID-19 [9]. Several studies [10,11] have prompted the immune-enhancing effect of probiotics to prevent or ameliorate viral infections. Interestingly, heat-treated, dead lactic acid bacteria were shown to have a significant antiviral effect [10]. Jung et al. reported that heat-treated *Lactobacillus casei* increased the survival rate of mice infected with influenza virus strain H3N2 and reduced the amount of virus in the lungs [12]. A study by Kobayasi et al. also demonstrated that mice administered heat-treated *Lactobacillus* strain b240 orally exhibited increased immunoglobulin expression and enhanced T-cell activity, resulting in increased resistance to the influenza virus [13]. In addition, heat-killed *L. plantarum* L-137 [14] and *L. casei* Shirota [15] were reported to have immunomodulatory activity through Th1 response.

Heat-treated *Lactiplantibacillus plantarum* nF1 (HT-nF1) is an inactivated form of *L. plantarum* nF1 isolated from Korean kimchi. Previous in vitro and in vivo studies revealed that activated immune cells induced the production of immunomodulatory substances, such as cytokines [16]. In addition, we confirmed that it has an immune-enhancing effect that induces the activity of NK cells and macrophages, resulting in an immunological defense function to protect the body from antigens such as pathogenic microorganisms [17]. Moreover, nF1 was effective at protecting mice from influenza virus attacks by activating anti-immune responses against three types of influenza viruses (A/H1N1, A/H3N2, and B/Yamagata) [18]. Food (yogurt), in which nF1 was added, had a protective effect against influenza virus infection by attenuating the innate immune system, evidenced by increased NK cell activity and cytokine production [19].

To confirm that these research results were similar in clinical study, we determined the effect of intake of HT-nF1 on immune function and safety in adult men and women with normal white blood cell (WBC) counts.

## 2. Materials and Methods

### 2.1. Study Subjects

Subjects were recruited from the Global Medical Research Center (Seoul, Korea), and 100 healthy adult volunteers were enrolled. All subjects were aged 19–75 years and the white blood cell (WBC) count in the peripheral blood of them was 3–8 × 10^3^ cells/μL, which is slightly lower than the normal number of WBCs in the blood. The exclusion criteria were as follows: (1) presence or history of disease (diabetes; cardiovascular system, including myocardial infarction or cerebrovascular disease within 6 months; immune system; respiratory system; liver biliary; kidney and urinary tract; nervous system; musculoskeletal disorders; psychic; infection and blood neoplasia; gastrointestinal symptoms, including heartburn or indigestion); (2) continuous consumption of antibiotics within the preceding 2 months of the initial visit; (3) presence or history of neurologically or psychologically important medial disease; (4) uncontrolled high blood pressure; (5) AST and ALT levels greater than three times the standard upper limit; (6) serum creatinine levels greater than two times the standard upper limit; (7) vaccination within one month prior to the screening test; (8) alcohol overuse and excessive smoking; (9) continuous consumption of probiotics (e.g., lactic acid bacteria), postbiotics (e.g., heat-killed probiotics), and prebiotics (e.g., dietary fiber, fructo-oligosaccharide) within the preceding month of the initial visit; (10) continuous consumption of dietary supplements within the preceding month of the initial visit; (11) participation in another clinical trial within the preceding month; and (12) pregnancy or lactation.

All subjects gave their informed consent for inclusion before they participated in the study. The study protocol was reviewed and approved by the Institutional Review Board (IRB) of the Global Medical Research Center, and the study was conducted after approval in October 2022 (IRB Number: GIRB-22O19-KQ) following the Declaration of Helsinki. The study protocol was registered in the International Clinical Trials Registry Platform of the WHO on 22 November 2022, with the following identification number: KCT0007927.

### 2.2. Study Design

The study was designed as a randomized, placebo-controlled, double-blind trial. After conducting a screening test and obtaining written informed consent, a total of 100 subjects were randomly assigned to a placebo or HT-nF1 group for 8 weeks of treatment. Randomization was carried out using a computer-generated random list. The block randomization method was used, in which the control group and test group were assigned 1:1, and the allocation ratio of men and women in each group was nearly equal. Because this study was conducted in a double-blind fashion, the assigned group of subjects was not disclosed to the researchers or subjects until the end of the study. Subjects were advised to take one capsule daily with plenty of water. For 8 weeks, the placebo group (*n* = 50) took a placebo, and the HT-nF1 (*n* = 50) group took capsules containing 5 × 10^11^ cells nF1. Food intake, Pittsburgh Sleep Quality Index, Global Physical Activity Questionnaire, Global Assessment of Recent Stress Scale, and Fatigue Severity Scale were recorded at baseline, 4 weeks, and 8 weeks.

### 2.3. Anthropometric Measurements and Blood Collection

Weight and body mass index (BMI) were measured. BMI was calculated as kilograms per square meter (kg/m^2^). The subjects were kept in a stable state for more than 10 min and their vital signs (pulse, blood pressure, and body temperature) were measured. After the subjects fasted for 12 h, venous blood samples were collected into regular tubes and EDTA-treated tubes. Blood samples were centrifuged to obtain serum and plasma and stored at −70 °C until analysis.

### 2.4. Isolation of Plasma and Peripheral Blood Mononuclear Cells (PBMCs)

PBMCs were separated from whole blood using density gradient centrifugation. Whole blood drawn from each participant was centrifuged at 1500× *g* for 10 min to obtain plasma and the buffy coat. After centrifugation, the plasma was transferred to fresh tubes. The buffy coat was mixed with phosphate-buffered saline (PBS) (Biowest, Nuaillé, France). The mixture of buffy coat and PBS was transferred to the top of Histopaque-1077 (Sigma Aldrich, St. Louis, MO, USA) and centrifuged at 1500× *g* for 15 min. Subsequently, the mononuclear cell layer was mixed with PBS and centrifuged at 2500× *g* for 15 min. The supernatant was removed, and the remaining cells were suspended in Roswell Park Memorial Institute 1640 medium (RPMI) (Biowest, Nuaillé, France), supplemented with 45% fetal bovine serum (Biowest, Nuaillé, France) and 10% dimethyl sulfoxide (Sigma Aldrich, St. Louis, MO, USA). The cells were then aliquoted, frozen, and stored in liquid nitrogen.

### 2.5. Cytotoxicity of NK Cells

NK cell-mediated cytotoxicity against K562 cells (Korean Cell Line Bank, Seoul, Korea) was assessed using the CytoTox 96 non-radioactive cytotoxicity assay kit (Promega Inc., Madison, WI, USA). Effector cells (PBMC) and target cells (2 × 10^4^ cells/well, K562 cells) were co-cultured in a 96-well plate at effector-to-target (E:T) cell ratios of 25:1, 10:1, and 5:1 and incubated for 4 h at 37 °C. After incubation, the plate was centrifuged at 250× *g* for 4 min. The supernatant was transferred to a fresh 96-well plate, and the CytoTox 96^®^ Reagent was added. Subsequently, they were incubated for 30 min at room temperature and protected from light. Finally, a stop solution was added and cytotoxicity was measured using a microplate reader (BioTek Instruments, Inc., Winooski, VT, USA) at 490 nm [20]. The cytotoxicity (%) was calculated using the following formula:

$$\%\;Cytotoxicity=\frac{Experimental-Effector\;Spontaneous-Target\;Spontaneous}{Target\;Maximum-Target\;Spontaneous}\times 100$$

### 2.6. Safety Parameter

Safety indicator tests were conducted as follows under a 12 h study period at baseline and week 8. Safety index evaluation was performed using hematological tests (RBC, Hb, Hct, MCV, MCH, MCHC, and PLT), differential cell counts (neutrophils, lymphocytes, monocytes, eosinophils, and basophils), blood chemical tests (AST, ALT, BUN, albumin, creatinine, eGFR, and TSH), and urine tests (specific gravity, pH, protein, glucose, ketone, bilirubin, urobilinogen, nitrite, erythrocyte, and leukocyte).

### 2.7. Statistical Analysis

The results were analyzed according to the intention-to-treat (ITT) principle. A normality test was performed on data distribution, and data with a non-normal distribution were converted to a normal distribution and analyzed. Statistical analysis was performed using the Statistical Analysis Systems package version 9.4 (SAS Institute, Cary, NC, USA), and statistical significance was defined as *p* < 0.05. In the case of continuous variables, the mean and standard error were presented, and in the case of functional evaluation, an estimate (β, change in the placebo group compared with the change in the test group) was also presented. In addition, the number of subjects was presented for categorical variables.

An intergroup comparison of subject characteristics at baseline was analyzed using a Student’s *t*-test for continuous variables and a Chi-square test or Fisher’s exact test for categorical variables. Intergroup comparison of compliance was analyzed using a Student’s *t*-test. Dietary intake and activity rate were analyzed using a linear mixed-effect model by considering group, time (week), and interaction between group and time (group × week) as random and fixed effects to analyze differences between groups according to intake period and comparison before and after within groups.

Functional evaluation variables were analyzed. To analyze differences between groups according to the intake period and comparison before and after within groups, a linear mixed effects model was used to analyze group, time (week), and the interaction between group and time (group × week). The WBC count at baseline and fat intake for 8 weeks, which had significant differences between groups, were corrected for analysis. In addition, values exceeding three times the interquartile range (less than Q1 − 3xIQR or greater than Q3 + 3xIQR) were considered outliers and excluded from the analysis. To confirm the effect of HT-nF1, the qualitative interaction trees (QUINT) package of R statistical software version 4.1.2, based on a machine learning algorithm, was used. The basic characteristics of the subjects (i.e., effect modifiers) that distinguish between subjects with a positive effect of HT-nF1 intake (responders) and those with no effect (non-responders) were selected. Based on this, the significance of the functional evaluation variables for each response and the non-response group was evaluated using a linear mixed-effects model.

Continuous variables among vital signs and clinical pathology tests were analyzed for differences between groups according to intake period and for comparison before and after within groups. Group, time (week), and the interaction between group and time (group × week) were considered as random and fixed effects and analyzed using a linear mixed-effect model. In addition, comparisons between groups of categorical variables during clinical pathological examination were analyzed using Fisher’s exact test, and comparisons before and after within groups were analyzed using McNemar’s test. Also, the occurrence, type, symptom severity, and causal relationship with the test food are described for adverse reactions, and comparisons between groups were performed using Fisher’s exact test.

## 3. Results

### 3.1. Subject Characteristics

**Figure 1:**
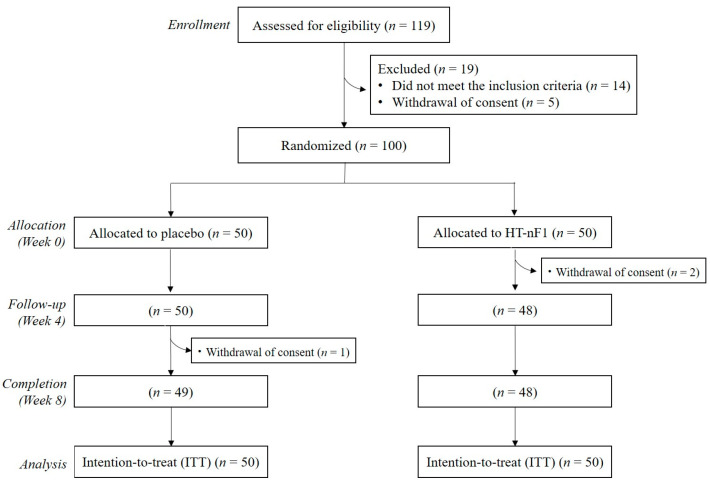
CONSORT diagram for the flow of subjects through the study. HT-nF1, heat-treated *Lactiplantibacillus plantarum* nF1.

The progress of this study is shown in Figure 1. A total of 119 people were screened, and 100 people who met the inclusion/exclusion criteria were randomly assigned to a control group (*n* = 50) and a test group (*n* = 50). Of 100 subjects, 2 (1 in the control group and 1 in the test group) were excluded because they stopped participating. Baseline characteristics are listed in Table 1. The average age was 38.5 ± 1.7 years for the placebo group and 40.2 ± 1.7 years for the HT-nF1 group, whereas the gender ratio was the same for both groups. There was a significant difference between the groups in WBC count, which was 5.1 ± 0.1 × 10^3^ cells/μL in the placebo group and 5.7 ± 0.2 × 10^3^ cells/μL in the HT-nF1 group (*p* = 0.010); however, differences in WBC may not be clinically significant because of changes within the normal range (4.0–10.0 × 10^3^ cells/μL). There was no significant difference between the groups for all other indicators. Compliance was above 100% on average for both groups, and there was no significant difference between groups.

### 3.2. Effects of HT-nF1 on Immune Markers

NK cell activity was measured based on E:T ratios of 25:1, 10:1, and 5:1 (Table 2). As shown in Table 2, there was no significant difference in NK cell activity measured at baseline between the placebo and HT-nF1 groups. Compared with the baseline, serum NK activity decreased in both groups at all E:T ratios at week 8. There was no significant difference in the effect on NK cell activity between the HT-nF1 group and the placebo group. Cytokine and immunoglobulin levels are listed in Table 2. After 8 weeks of intake, a change in IL-12 was significantly increased in the HT-nF1 group compared with the change in the placebo group (β = 0.6, *p* = 0.045). Compared with the baseline, serum IL-12 levels were significantly decreased in the placebo group at week 8 (*p* = 0.007). Other indicators (IL-10, TNF-α, and slgA) were not significantly different between the HT-nF1 and placebo groups.

### 3.3. Effects of HT-nF1 on Responders and Non-Responders

We confirmed the effects of HT-nF1 using the QUINT package based on a machine-learning algorithm. To analyze the responders and non-responders following intake of HT-nF1, two criteria at baseline were selected as effect modifiers: secretory immunoglobulin A (sIgA) and NK cell activity (E:T = 10:1). Based on these factors, the effect in the HT-nF1 group compared with the placebo was confirmed.

**Figure 2:**
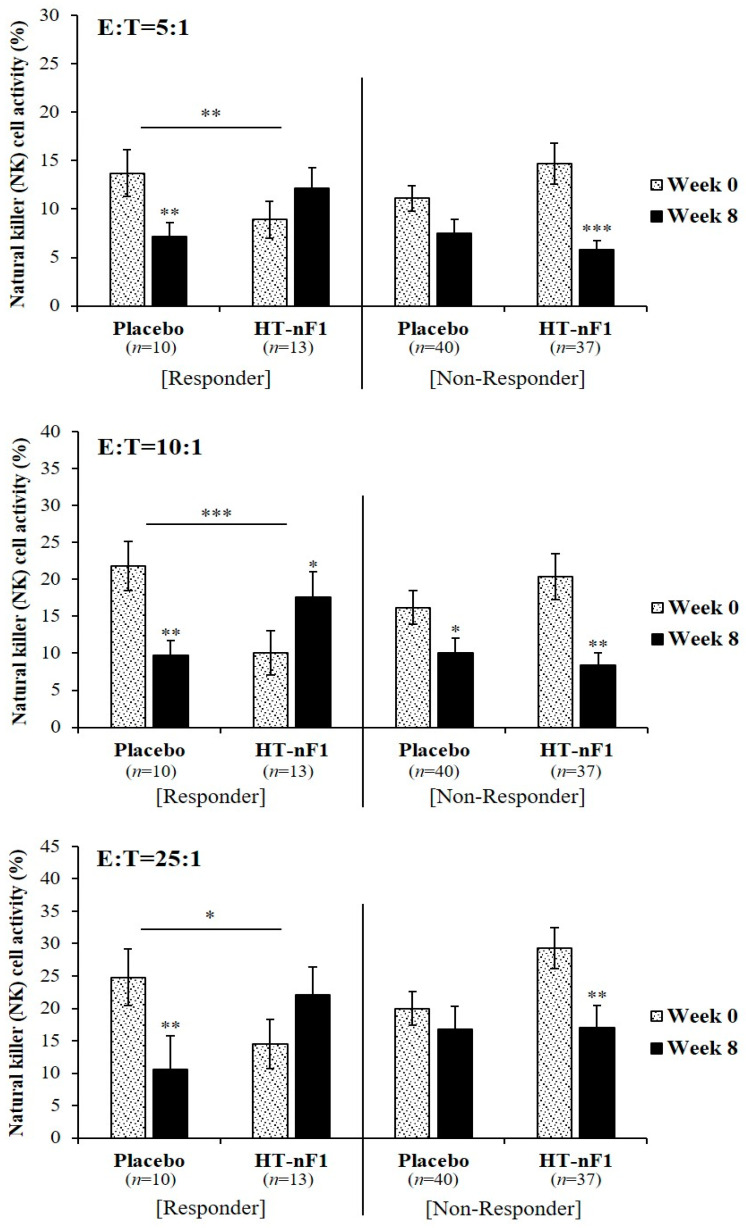
NK cell activity through stratified analysis according to sIgA in responders (≤49.61 μg/mL) and non-responders (>49.61 μg/mL) to HT-nF1. NK cell, natural killer cell; HT-nF1, heat-treated *Lactiplantibacillus plantarum* nF1; sIgA, secretory immunoglobulin A; E, effector cell; T, target cell. The estimate and *p*-value were from a linear mixed-effect model to compare the changes for 8 weeks between the groups. WBC at baseline and dietary fat intake for 8 weeks were adjusted. * *p* < 0.05; ** *p* < 0.01; *** *p* < 0.001.

A stratified analysis was performed based on sIgA (Figure 2). In subjects with sIgA levels ≤49.61 μg/mL (responder) (placebo, *n* = 10; HT-nF1, *n* = 13), NK cell activity was reduced in the placebo group after 8 weeks from baseline. However, NK cell activity increased in the group that consumed HT-nF1 for 8 weeks. At a concentration ratio of E:T = 5:1, when HT-nF1 was ingested for 8 weeks, it increased by approximately 1.3-fold to 12.2 ± 2.1% compared with before ingestion. In the case of E:T = 10:1, it increased significantly to 17.6 ± 3.4%. Even at a concentration of E:T = 25:1, ingestion of HT-nF1 increased NK cell activity by approximately 1.5-fold to 22.1 ± 4.3%. Furthermore, we compared the change in NK cell activity when placebo and HT-nF1 were each consumed for 8 weeks. The results indicated a change in NK cell activity in the HT-nF1 group at concentrations of E:T = 5:1, 10:1, and 25:1, which were significantly increased compared with the change in NK cell activity in the placebo (E:T = 5:1, β = 9.2, *p* = 0.004; E:T = 10:1, β = 20.5, *p* < 0.001; E:T = 25:1, β = 18.4, *p* = 0.011). For cases in which sIgA was high (>49.61 μg/mL), there was no significant change in NK cell activity.

**Figure 3:**
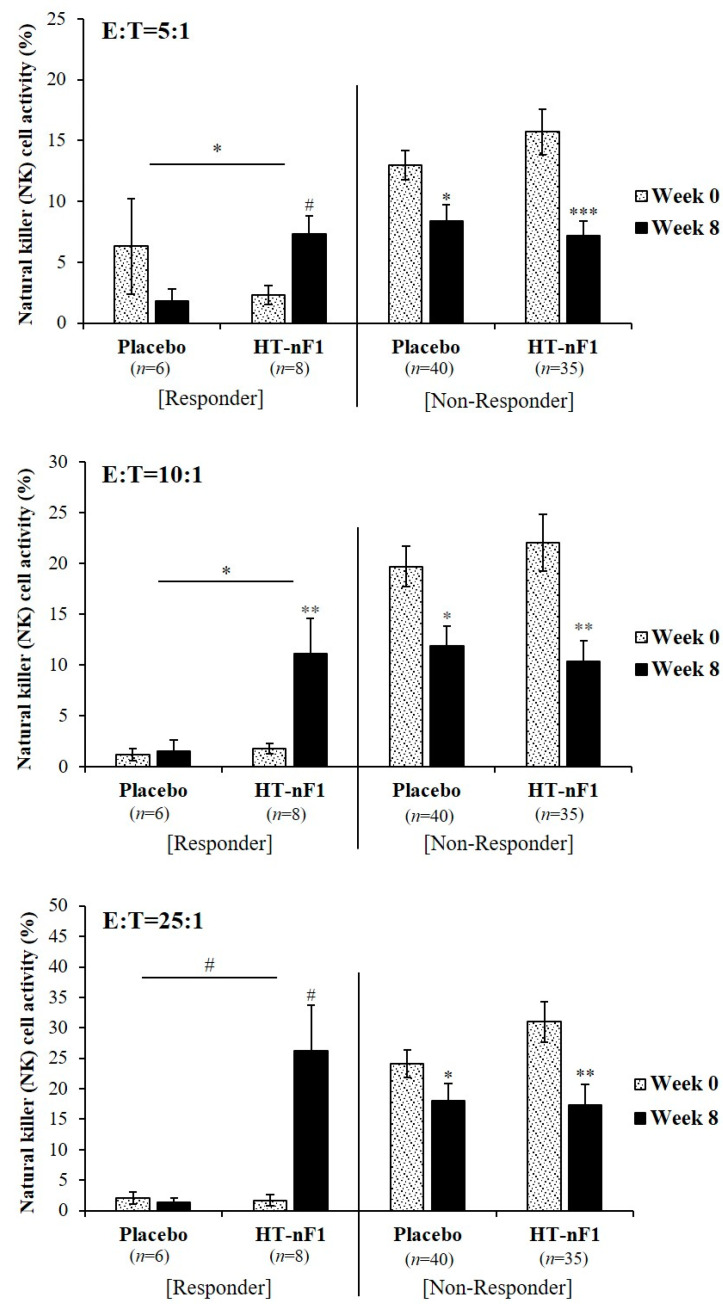
NK cell activity through stratified analysis according to NK cell activity (E:T = 10:1) in responders (≤3.59%) and non-responders (>3.59%) to HT-nF1. HT-nF1, heat-treated *Lactiplantibacillus plantarum* nF1; E, effector cell; T, target cell. The estimate and *p*-value were from a linear mixed-effect model to compare the changes for 8 weeks between the groups. WBC at baseline and dietary fat intake for 8 weeks were adjusted. Not detected was excluded; placebo (*n* = 4), HT-nF1 (*n* = 7). * *p* < 0.05; ** *p* < 0.01; *** *p* < 0.001; # *p* < 0.1.

The results based on NK cell activity (E:T = 10:1) (Figure 3) also indicated that in subjects with low NK cell activity (≤3.59%, responder) (placebo, *n* = 6; HT-nF1, *n* = 8), it has increased when HT-nF1 was consumed for 8 weeks. At E:T = 5:1 and 10:1 concentration ratios, the NK cell activity of the HT-nF1 group was 7.3 ± 1.5% and 11.1 ± 3.5%, respectively, which significantly increased by approximately 3.1- and 6.1-fold (E:T = 5:1, *p* = 0.083; E:T = 10:1, *p* = 0.003) after 8 weeks of treatment. At a ratio of E:T = 25:1, the NK cell activity of the placebo group decreased, but when HT-nF1 was consumed for 8 weeks, it increased approximately 15-fold to 26.2% ± 7.5% (*p* = 0.071). The change in NK cell activity when HT-nF1 was ingested for 8 weeks compared with the change in NK cell activity when the placebo was ingested for 8 weeks, a significant increase was observed for HT-nF1 at all ratios (E:T = 5:1, β = 12.2, *p* = 0.041; E:T = 10:1, β = 9.3, *p* = 0.037; E:T = 25:1, β = 17.9, *p* = 0.086). Consistently, subjects with high NK cell activity (>3.59%, non-responder) showed no significant change.

### 3.4. Safety Analysis

To analyze the safety of HT-nF1, hematological and blood biochemical analyses were performed at baseline and week 8 (Table 3). There were significant differences in some indicators when comparing before and after intake between groups and within groups, but all changes were within the normal range and there were no clinically significant changes. No serious adverse reactions related to the test food were observed, and there were no significant differences between groups in terms of occurrence, type, symptom severity, or causal relationship with the test food.

## 4. Discussion

Immune regulation in the human body occurs through the processes that are promoted and suppressed, and if immune regulation fails, diseases or other problems can occur [21]. In addition, interest in improving human immunity has increased because of various factors, such as the prevalence of infectious diseases and the aging population. The demand for immune-enhancing functional materials is also steadily increasing [22,23,24]. Recent studies have indicated that probiotics are associated with immunomodulatory effects [25,26,27]. Chong et al. [25] evaluated the effect of *L. plantarum* DR7 on upper respiratory tract infection and confirmed that NK cell activity was enhanced in subjects who consumed *L. plantarum* DR7 compared with the control group. *L. plantarum* YU regulates immune function through activation of the Th1 immune response and lgA production [26]. In addition, probiotics, such as *L. casei* Shirota, *L. acidophilus* X37, and *B. bifidum* MF 20/5, increase innate immunity by increasing NK cell activity [27]. Postbiotics are inactivated bacterial cells that provide health benefits to humans, which include non-living bacteria, cell walls, and metabolites. They have similar effects as probiotics but are used in patients with weakened immune systems, which has been reported recently [28,29,30,31]. Postbiotics are associated with immunomodulation and strengthening the innate and adaptive immune systems [32,33]. Heat-treated *L. sakei* KU15041 and *L. curvatus* KU15003 regulate immunity by increasing the expression of TNF-α, IL-1β, and IL-6 in RAW 264.7 cells [34]. Heat-killed *L. casei* IMAU60214 improved immune function by regulating macrophages in malnourished children [35]. When heat-treated *L. plantarum* LM1004 was consumed for 8 weeks, it stimulated innate immunity and significantly increased NK cells and IL-12 levels [36]. Hirose et al. [37] suggested that the daily consumption of heat-killed *L. plantarum* L-137 increases Th1 immunity and confirmed that it was safe, even when consumed at high doses and for long periods [38]. In addition, in many studies [39,40,41], *Lactobacillus* increased the levels of Th1 cytokines (e.g., IL-12, IFN-r). However, since it did not affect the production of Th2 cytokines (e.g., IL-4, IL-5), it is thought to enhance immunity through the Th1 mechanism.

This study was performed to evaluate the effect of 8 weeks of consumption of heat-treated *L. plantarum* nF1 (HT-nF1) on improving immunity. NK cells play an important role in innate immune responses [42]. They also regulate immune responses by producing cytokines, which can activate various cell types involved in the adaptive and innate immune systems [6,43,44]. Depending on the type of cytokine, Th1 cells and Th2 cells are converted into lymphocytes. Th1, which induces a normal immune response, is an inflammatory response that involves TNF-α, IL-2, IL-12, and IFN-γ. Th2 induces an autoimmune response and activates humoral immunity by producing IL-4, IL-5, IL-6, and IL-10 [45,46]. Increased IL-12 production is associated with increased cellular immunity and phagocytic function [47]. IL-12 is also important for inducing Th1 immunity [48] and directly activates CD56+ NK cell-mediated cytotoxicity [49]. IL-12 affects NK cell regulation [50]. The results of the present study confirmed that a change in IL-12 was significantly increased in the test group compared with the control group in all subjects (β = 0.6, *p* = 0.045). NK activity may also be increased through a significant increase in IL-12; however, intake of HT-nF1 did not affect NK activity in all subjects (Table 2). Because this study was conducted from November to March, most of the subjects may have experienced cold stress [51,52,53,54] due to the winter weather and sudden temperature changes [55]. In addition, in the case of female subjects, immune-related indicators may be affected by physiological and general environmental conditions, such as the menstrual cycle [37,56,57]. However, we confirmed that the intake of HT-nF1 had a positive effect on improving immunity in subjects with low slgA and NK activity, that is, with a relatively weak immune system (Figure 2 and Figure 3).

There are various types of immunoglobulins that play an important role in immunity, such as IgG, IgM, IgA, and IgE [58]. Of these, IgA has a primary defense role against pathogens and activates mucosal immunity [59,60]. Individuals with low slgA levels are readily exposed to various diseases, such as respiratory infections [61,62]. Probiotics and postbiotics can regulate immunity by increasing the secretion of slgA [63,64,65,66,67]. The consumption of milk containing *L. rhamnosus* HN001 or *Bif. lactis* HN019 increased NK activity in elderly people over 70 years of age [68]. NK activity also increases when healthy elderly people take probiotic supplements [69,70,71]. In addition, in a double-blind, placebo-controlled clinical study, heat-killed *L. gasseri* TMC0356 regulated immunity by significantly increasing the number of CD8 + T cells [72]. Moreover, an increase in NK cell activity was observed in the splenocytes of mice orally administered a postbiotic complex containing *L. plantarum* during immunosuppression [33]. Therefore, our results suggest that nF1 intake maintains the immune status by activating NK cells in subjects with relatively weak immune systems.

Previous studies showed that nF1 induced spleen cell proliferation and promoted a Th1 response and cytokine (IL-12, TNF-α, and IFN-γ) production [16,41]. nF1 increased the production of TNF-α, IL-2, and IL-6 in RAW 264.7 macrophages, which resulted in the activation of NF-κB and phosphorylation of IκBα [17]. In addition, nF1 intake improves immune function by increasing the levels of total IgG, TNF-α, IL-2, and IL-12 in the serum of cyclophosphamide (CPP) immunosuppressed mice [16,17]. Lee et al. [73] demonstrated that immunity was regulated by increasing the level of total IgA in mice with colon cancer induced by azoxymethane (AOM)/dextran sodium sulfate (DSS) exposure. In humans, nF1 regulates immune function [46,74]. In IBS patients, subjects who consumed kimchi containing nF1 showed regulated immune function with modulated levels of cytokines (TNF-α, IL-12) in the serum [74]. Moreover, when healthy older adults consumed yogurt containing nF1 (heat-treated *L. plantarum* nF1, *L. paracasei*, and *B. lactis*) for 12 weeks, NK cell activity and the concentration of IFN-γ and IgG1 increased, thus improving immune function [46]. In addition, the effect of regulating immune function was confirmed when nF1 was added to food [19,46,75]. When yogurt containing nF1 was consumed by mice infected with influenza A virus (IAV), NK cell activity increased [19]. NK cell activity was also increased in mice that consumed kimchi containing nF1 [75]. Therefore, it is thought that the nF1 immune modulation is effective based on the Th1 response. In the present study, NK cell activity significantly increased in subjects with low slgA and NK cell activity (E:T = 10:1). These results suggest that nF1 strengthens and regulates the immune system and restores normal immunity in people with weak immune systems (e.g., children [16,76], elderly [16,46], and patients [74]).

In the safety evaluation, the frequency of adverse events and blood levels were considered negligible as they occurred within the normal or reference range. Therefore, heat-treated *Lactiplantibacillus plantarum* nF1 not only has a positive effect on regulating immunity but is also a safe material for humans, thus confirming its potential as a raw material that can help improve immunity. 

## 5. Conclusions

In this study, we examined the effect of nF1 for 8 weeks on immune function in a clinical study. A change in IL-12 was significantly increased in the HT-nF1 group compared with the placebo group, and the change in NK cell activity was significantly increased in subjects with low slgA and subjects with low NK cell activity, which indicates that nF1 intake was more effective. Therefore, intake of nF1 may be an effective strategy to improve immune function.

## Figures and Tables

**Table 1:** Baseline characteristics of intention-to-treat (ITT) subjects who participated in this study ^(1)^.

Variables	Placebo (*n* = 50)	HT-nF1 (*n* = 50)	*p*-Value ^(2)^
Age (year)	38.5 ± 1.7	40.2 ± 1.7	0.492
Gender (male/female)	16/34	16/34	1.000
Menstruation (Y/N/NA)	29/5/16	26/8/16	0.652
Alcohol drinker (Y/N)	29/21	22/28	0.161
Alcohol amount (SD/week)	0.04 ± 0.03	0.29 ± 0.19	0.191
Smoker (Y/N)	2/48	4/46	0.678
Smoking amount (cigarettes/d)	0.1 ± 0.1	0.3 ± 0.2	0.383
BMI (kg/m^3^)	23.4 ± 0.4	23.2 ± 0.4	0.698
Natural killer cell activity (%)			
E:T = 5:1	11.7 ± 1.1	13.3 ± 1.7	0.430
E:T = 10:1	17.3 ± 2.0	18.2 ± 2.6	0.761
E:T = 25:1	20.9 ± 2.3	26.4 ± 3.1	0.155
WBC (10^3^/μL)	5.1 ± 0.1	5.7 ± 0.2	0.010

^(1)^ Mean ± SE (all such values). HT-nF1, heat-treated *Lactiplantibacillus plantarum* nF1; Y, yes; N, no; NA, not applicable; SD, standard drink; BMI, body mass index; E, effector cell; T, target cell; WBC, white blood cell. ^(2)^ Student’s *t*-test for continuous variables, and Chi-square test or Fisher’s exact test for categorical variables were used to compare differences between the groups.

**Table 2:** Natural killer cell activity, serum cytokine, and immunoglobulin levels at the baseline and endpoint in the placebo and HT-nF1 groups ^(1)^.

Variables	Placebo (*n* = 50)		HT-nF1 (*n* = 50)		Estimate ^(2)^	*p*-Value ^(2)^
	Baseline	Week 8	Baseline	Week 8		
NK cell activity (%)						
E:T = 5:1	11.7 ± 1.1	6.7 ± 0.8 **	13.3 ± 1.7	7.1 ± 0.9 ***	−1.0	0.652
E:T = 10:1	17.3 ± 2.0	9.0 ± 1.3 **	18.2 ± 2.6	10.3 ± 1.6 **	1.2	0.732
E:T = 25:1	20.9 ± 2.3	15.2 ± 2.5 *	26.4 ± 3.1	18.2 ± 2.8 **	−2.0	0.649
IL-10 (pg/mL)	2.4 ± 0.2	2.4 ± 0.2	2.9 ± 0.4	3.1 ± 0.4	0.0	0.979
IL-12 (pg/mL)	1.6 ± 0.3	1.0 ± 0.1 **	1.0 ± 0.1	1.0 ± 0.1	0.6	0.045
TNF-α (pg/mL)	3.4 ± 0.4	3.9 ± 0.5	4.3 ± 0.6	4.2 ± 0.6	−0.5	0.523
slgA (μg/mL)	74.7 ± 4.5	71.4 ± 4.5	81.3 ± 5.3	78.4 ± 5.3	−0.9	0.888

^(1)^ Mean ± SE (all such values). HT-nF1, heat-treated *Lactiplantibacillus plantarum* nF1; E, effector cell; T, target cell; IL, interleukin; TNF, tumor necrosis factor; sIgA, secretory immunoglobulin A. ^(2)^ Estimates and *p*-values were from a linear mixed-effect model to compare the changes for 8 weeks between the groups. WBC at baseline and dietary fat intake for 8 weeks were adjusted. * *p* < 0.05, ** *p* < 0.05, *** *p* < 0.05 different from baseline.

**Table 3:** Hematological and blood chemistry tests at the baseline and endpoint in the placebo and HT-nF1 groups ^(1)^.

Variables	Placebo (*n* = 50)		HT-nF1 (*n* = 50)		*p*-Value ^(2)^		
	Baseline	Week 8	Baseline	Week 8	Group	Week	Group × Week
Hematological test							
RBC (10^6^/μL)	4.6 ± 0.1	4.6 ± 0.1	4.6 ± 0.1	4.5 ± 0.1 **	0.391	0.061	0.043
Hemoglobin (g/dL)	13.8 ± 0.2	13.7 ± 0.2	13.8 ± 0.2	13.5 ± 0.2 **	0.814	0.020	0.064
Hematocrit (%)	41.6 ± 0.6	41.4 ± 0.6	41.8 ± 0.5	40.6 ± 0.5 **	0.680	0.006	0.045
Platelet (10^3^/μL)	287.8 ± 9.9	287.7 ± 9.8	275.3 ± 8.8	279.9 ± 7.7	0.379	0.727	0.661
Neutrophils (%)	55.8 ± 1.2	54.6 ± 1.3	55.9 ± 1.3	52.5 ± 1.2 **	0.525	0.006	0.187
Lymphocytes (%)	33.3 ± 1.0	34.7 ± 1.2	34.5 ± 1.3	37.2 ± 1.2 *	0.219	0.005	0.402
Monocytes (%)	6.7 ± 0.3	6.8 ± 0.2	6.7 ± 0.2	7.1 ± 0.2	0.735	0.052	0.435
Eosinophils (%)	3.5 ± 0.5	3.1 ± 0.3 *	2.3 ± 0.3	2.5 ± 0.2	0.055	0.473	0.038
Basophils (%)	0.8 ± 0.1	0.8 ± 0.1	0.7 ± 0.1	0.7 ± 0.0	0.318	0.531	0.409
Blood chemistry test							
AST (U/L)	20.9 ± 0.8	21.3 ± 1.0	22.6 ± 0.9	23.2 ± 1.3	0.129	0.493	0.883
ALT (U/L)	18.5 ± 1.6	19.4 ± 1.8	20.1 ± 1.8	22.1 ± 2.3	0.339	0.242	0.609
BUN (mg/dL)	12.1 ± 0.6	12.2 ± 0.5	12.5 ± 0.5	12.6 ± 0.5	0.624	0.871	0.837
Albumin (g/dL)	4.5 ± 0.0	4.4 ± 0.0	4.5 ± 0.0	4.5 ± 0.0 *	0.483	0.007	0.829
Creatinine (mg/dL)	0.71 ± 0.02	0.72 ± 0.02	0.71 ± 0.02	0.71 ± 0.02	0.908	0.292	0.257

**^(^**^1)^ Mean ± SE (all such values). HT-nF1, heat-treated *Lactiplantibacillus plantarum* nF1; RBC, red blood cell; AST, aspartate aminotransferase; ALT, alanine aminotransferase; BUN, blood urea nitrogen. ^(2)^ A linear mixed-effect model to compare the changes after 8 weeks between the group, week, and group × week. * *p* < 0.05; ** *p* < 0.01 different from baseline.

## Data Availability

Data are included in the article.
